# Chinese Adaptation of the Nurses Time‐Wasters Questionnaire: Reliability and Validity Testing

**DOI:** 10.1155/jonm/2162912

**Published:** 2026-08-17

**Authors:** Ruixue Wang, Yu Han, Jiao Zhang, Cuiping Ni, Kui Fang

**Affiliations:** ^1^ Department of Neurosurgery, The First Affiliated Hospital of China Medical University, Shenyang, Liaoning, China, cmu.edu.cn; ^2^ School of Nursing, China Medical University, Shenyang, Liaoning, China, cmu.edu.tw; ^3^ First Department of Infectious Diseases, The First Affiliated Hospital of China Medical University, Shenyang, Liaoning, China, cmu.edu.cn

**Keywords:** nurses, psychometrics, reliability, time wasters, time-wasting, validity

## Abstract

**Introduction:**

Effective time management is crucial for nurses, and identifying factors contributing to time‐wasting behaviors is a key component of time management. Currently, China lacks validated, standardized tools for assessing time wasters among nurses. This study is designed to adapt the Nurses Time‐Wasters Questionnaire into a Chinese version and to evaluate the reliability and validity of this adapted instrument within a population of Chinese nurses.

**Methods:**

This cross‐sectional study was conducted in China from September to November 2025, recruiting 330 nurses through convenience sampling. The translation process used the forward–backward translation method. Content validity was assessed by consulting 10 experts and calculating I‐CVI and S‐CVI/Ave values. Construct validity was assessed using exploratory factor analysis (EFA; *n* = 130) followed by confirmatory factor analysis (CFA; *n* = 200). Internal consistency reliability was measured with Cronbach’s α coefficient. All analyses were conducted in SPSS 27.0 and AMOS 29.0.

**Results:**

All items were retained, with only some items undergoing cultural adaptation modifications. Adequate sampling was indicated by the Kaiser–Meyer–Olkin (KMO) score of 0.960. The data’s eligibility for factor analysis was confirmed by the significant Bartlett’s test of sphericity (*χ* ^2^ = 1202.469, df = 78, *p* < 0.001). One factor that explained 63.481% of the total variance was identified using EFA. The single‐factor model showed good fit using CFA (*χ* ^2^/df = 1.226, RMSEA = 0.034, GFI = 0.942, and TLI = 0.991). The total questionnaire’s Cronbach’s alpha coefficient was 0.956.

**Conclusion:**

The Chinese version of the Nurses Time‐Wasters Questionnaire demonstrates good reliability and validity, making it a reliable and effective measurement tool for assessing time‐wasting behaviors among clinical nurses in China.

## 1. Introduction

Time management is the process of systematically organizing and allocating time to improve productivity and achieve personal and professional goals [1]. A meta‐analysis examining the effectiveness of time management revealed its significant role across various contexts, demonstrating that it can enhance work performance [2]. Therefore, time management is important for nurses. It not only affects their work efficiency but also directly impacts their ability to manage role conflicts and maintain work‐life balance, ultimately influencing their capacity to provide high‐quality care [3]. In high‐intensity healthcare environments, nurses must balance various obligations, such as administering medications, monitoring patients’ vital signs, updating medical records, collaborating with other healthcare workers, and providing emotional support to patients and their families [4]. Effective time management enables nurses to effectively allocate time and resources, ensuring that patients’ needs are met without affecting the quality of care. It assists nurses in organizing the order of duties and prioritizing critical tasks, hence lowering the incidence of errors, which can have major consequences for patient safety [4]. Furthermore, scientific time management can improve patient satisfaction because nurses are able to execute their obligations more efficiently and with less stress [5].

Time management encompasses a variety of behaviors aimed at allocating and organizing time efficiently [6]. Beyond setting goals, prioritizing tasks, and planning, identifying strategies to reduce time wasters is a key component of time management [7]. Nurses face ongoing challenges as they manage an increasing number of complex tasks and high‐intensity work conditions within limited time frames [8]. Time wasters are significant challenges faced by nurses. Therefore, identifying time‐wasting behaviors has become a critical component of time management. The consequences of wasting time include inefficient work performance, reduced productivity, and the potential to overlook important nursing tasks [9]. Several studies have identified gossiping, taking phone calls and socializing [10], and using paper documents [11, 12] as time‐wasting activities. With the advancement of healthcare systems, Hospital Information System (HIS) has been widely adopted in hospitals, yet it also faces numerous challenges [13], such as system response time [14].

A study on the causes of nurses’ time wasters discovered that the positional features of a specific ward influenced the nurses’ moves between wards, as well as the frequency with which the nurses entered the patients’ rooms or the nurses’ station [11]. These findings are concerning. The time‐wasting behavior of nurses, on the one hand, will reduce the time invested in patients, thereby affecting the satisfaction of medical staff as well as patient education and health promotion activities, which may lead to incorrect prescriptions and treatment errors [15]. On the other hand, it also reduces nurses’ work efficiency, occupies their non‐working hours, and increases their work pressure and professional burnout. Therefore, it is crucial for nurses to manage their time effectively and minimize time wasters.

Given the critical importance of reducing time wasters among nurses, an efficient and reliable instrument to evaluate nurses’ time wasters is obviously needed. However, in China, there are relatively few studies on the factors of nurses’ time wasters. More attention is paid to time management [16], and it mainly focuses on adolescents [17]. There is a lack of reliable evaluation tools for the factors of time wasters. In 2024, Palestinian author Zyoud [8] developed a Nurses Time‐Wasting Scale comprising three dimensions and 11 items to assess factors contributing to nurses’ time‐wasting behaviors. In 2025, another Palestinian author, Qtait, created the Nurses Time‐Wasters Questionnaire to evaluate time‐wasting factors faced by hospital nurses [18]. Zyoud’s [8] scale focuses more on time‐wasting behaviors generated by individual nurses. Qtait’s questionnaire not only evaluates individual behavioral factors but also assesses external hospital environmental factors. Therefore, Qtait’s questionnaire is more comprehensive than Zyoud’s scale.

To our knowledge, suitable tools for assessing time‐wasting factors among Chinese nurses remain limited. Consequently, we lack a comprehensive understanding of the factors contributing to time wasters among Chinese nursing staff. This study intends to fill this gap by translating, modifying, and validating the Nurses Time‐Wasters Questionnaire [18] for the Chinese environment while evaluating its validity and reliability. This will provide an effective tool for evaluating time‐wasting factors among nurses, thereby enhancing work efficiency, improving the quality of nursing services, and offering valuable guidance for nursing managers in decision‐making.

## 2. Methods

### 2.1. Aim

The purpose of this study is to assess the psychometric properties of the Nurses Time‐Wasters Questionnaire in Chinese.

### 2.2. Research Design

This study employed a cross‐sectional design and was conducted in China from September to November 2025.

### 2.3. Study Participants

Convenience sampling was used to select participants from 12 Chinese hospitals. The participating nurses were chosen from a variety of hospital departments, including internal medicine, surgery, obstetrics and gynecology, and pediatrics, in order to improve the scale’s generalizability. The sample size should be 10–20 times the scale’s item count due to factor analysis requirements [19]. The calculated sample size was *n* = [(10–20) × 13] ÷ (1%–10%) ≈ 144–289, taking into account a 10% invalid answer rate [20]. Ultimately, 330 valid responses were collected from 347 questionnaires, yielding an effective response rate of 95.1%. According to methodological guidelines, exploratory factor analysis (EFA) usually needs 2 to 20 individuals per item [21], whereas confirmatory factor analysis (CFA) needs a sample size between 150 and 500, with a minimum of 200 participants advised [22, 23]. The sample was divided into two groups at random: 200 for CFA (in compliance with the minimum needed sample size) and 130 for EFA (10 participants per item). Dividing a sample at random is the most straightforward method of dividing it in half [24, 25]. Inclusion criteria were willingness to participate, registered nurses in China, and at least a year of clinical experience. Participants who withdrew midway through the questionnaire completion process were excluded.

### 2.4. Data Collection

Prior to data collection, the researcher communicated with the nursing administration departments of multiple hospitals to clarify the study’s objectives and significance and to secure their approval. Following authorization, eligible nurses were informed about the research and asked to provide written informed consent. Subsequently, an electronic questionnaire was developed via the Wenjuanxing platform and disseminated through WeChat. The questionnaire’s opening page contained comprehensive instructions to help participants complete it correctly.

### 2.5. Study Instruments

#### 2.5.1. Demographic Questionnaire

Participants’ age, gender, marital status, education level, work experience, workplace, and work units were all gathered using the demographic questionnaire.

#### 2.5.2. Developing the Chinese Version of the Nurses Time‐Wasters Questionnaire (NTWQ‐C)

The time‐wasters questionnaire for nurses was developed by Mohammad Qtait [18] and comprises 13 items. It is a 5‐point Likert scale (never = 1, rarely = 2, sometimes = 3, very often = 4, and always = 5). The responses’ mean scores were as follows: 1.79 for “never” (very low), 1.8–2.59 for “rarely” (low), 2.6–3.39 for “sometimes” (medium), 3.4–4.19 for “very often” (high), and 4.2–5 for “always” (very high). The Cronbach’s alpha reliability score of 0.890 for the entire scale showed that the study items had a high degree of consistency.

Prior to the study, we contacted the original author to request permission to translate the time‐wasters questionnaire into Chinese. The cross‐cultural adaptation process of the nurses’ time‐wasters questionnaire was conducted following the guidelines outlined by Beaton [26]. Below is the process of localizing the questionnaire into Chinese.

Firstly, two researchers, fluent in English and having passed the CET‐6 for nursing graduates, independently translated the nurses’ time‐wasters questionnaire into Chinese, leading to versions S1 and S2.

Second, when the inconsistencies between S1 and S2 were synthesized and resolved, the original NTWQ‐C (S3) was created.

Thirdly, S3 was independently back‐translated into English versions W1 and W2 by a nursing PhD candidate with clinical experience and an English PhD candidate, without them having seen the original English. After the back‐translation was complete, the original scale and the back‐translated versions W1 and W2 were examined by a clinical nurse with international experience to make sure the Chinese translation faithfully captured the original questionnaire. Following a detailed comparison and proofreading process, the W3 questionnaire in Chinese was finalized to achieve semantic, idiomatic, experiential, and conceptual equivalence.

Fourth, a ten‐person expert committee was formed to help with W3’s cultural adaptation. This committee comprised four PhD holders, four individuals with postgraduate degrees, and two with graduate degrees, representing a range of specialties in clinical medicine, nursing management, clinical nursing, and nursing education. The questionnaire has been modified to better fit the nursing and cultural setting in China with the help of experts with five to 20 years of experience. The researchers finalized the NTWQ‐C with 13 items after making changes and enhancements to some scale items based on the expert panel’s recommendations.

Fifthly, based on the study’s inclusion and exclusion criteria, 25 nurses were chosen to pretest the NTWQ‐C. The group included 22 female and 3 male postgraduate students, each with 2–5 years of work experience. Some of them offered suggestions regarding the language of the two items. The researchers discussed the suggestions, which were then adopted and adjusted to develop the NTWQ‐C.

### 2.6. Statistical Analysis

Exploratory and CFAs were performed using SPSS Version 27.0 and AMOS Version 29.0. The data distribution was presented using descriptive statistical analyses, and two‐tailed significance tests with a significance threshold of *p* < 0.05 were carried out.

### 2.7. Ethical Considerations

This study was approved by the Ethics Committee of the First Affiliated Hospital of China Medical University (NO. 2025–595) and it followed the Declaration of Helsinki’s ethical guidelines.

### 2.8. Validity Analysis

#### 2.8.1. Content Validity

The NTWQ‐C’s content validity was tested using the Content Validity Index (CVI), which includes both the I‐CVI and the S‐CVI. Ten experts from the same panel that participated in the cultural adaptation stage were asked to rate the relevance of each item on a 4‐point Likert scale (1 = irrelevant, 2 = weakly relevant, 3 = strongly relevant, and 4 = very strongly relevant). The I‐CVI was determined by dividing the number of experts who rated the item 3 or 4 by the total number of experts. I‐CVI > 0.78 and S‐CVI/Ave ≥ 0.90 were considered satisfactory [27].

#### 2.8.2. Structural Validity

The structural validity of the NTWQ‐C was evaluated using EFA and CFA. Initially, the Kaiser–Meyer–Olkin (KMO) measure and Bartlett’s sphericity test were used to determine whether the data were suitable for EFA. Data met criteria for EFA, including a KMO value above 0.7 and a significant *χ* ^2^ result from Bartlett’s test (*p* < 0.05) [28]. EFA utilized principal component analysis and varimax rotation, extracting factors with eigenvalues equal to or greater than 1.0. A factor loading of 0.4 or above was the threshold for item retention, and items with loadings below this or with cross‐loadings were discarded [29].

The key indices for evaluating model fit in CFA are: (1) chi‐square/degree of freedom ratio (*χ* ^2^/df < 3); (2) Goodness of Fit Index (GFI > 0.900); (3) Root Mean Square Error of Approximation (RMSEA < 0.100); (4) Comparative Fit Index (CFI > 0.900); (5) Tucker–Lewis Index (TLI > 0.900); and (6) Incremental Fit Index (IFI > 0.900) [30, 31].

Convergent validity was further evaluated using the standardized factor loadings from the CFA, the average variance extracted (AVE), and the composite reliability (CR). An AVE ≥ 0.50 and a CR ≥ 0.70, with standardized loadings ≥ 0.50, were taken to indicate satisfactory convergent validity [32, 33].

#### 2.8.3. Assessing Reliability

This study employed Cronbach’s alpha coefficient to assess the internal consistency reliability of the scale [34]. Cronbach’s alpha ranges from 0 to 1, with values exceeding 0.70 indicating acceptable reliability and those above 0.80 demonstrating good reliability [35–37].

## 3. Results

Due to linguistic and cultural considerations, certain items in the questionnaire were modified. Based on consensus discussions among experts, cultural adaptations were made to items 1–3, 5, 6, and 10–13 (see S3 for detailed item‐by‐item modifications, including original items, translated items, final Chinese versions, and rationales).

### 3.1. Participant Characteristics

A total of 330 nurses were recruited to participate in this study and provided informed consent. Of these, 97.9% were women and 2.1% were men. The majority of nurses (75.5%) were married and worked in internal medicine (52.1%) or surgery (25.8%). The majority (89.7%) have a bachelor’s degree. Approximately 71.5% have over 10 years of experience. Table 1 shows other characteristics of the individuals.

**TABLE 1:** The demographic characteristics of nurses.

Variable	Category	*n*	Percentage (%)
Gender	Male	7	2.1
	Female	323	97.9

Age (years)	18–25	33	10.0
	26–30	38	11.5
	31–40	164	49.7
	≥ 41	95	28.8

Marital status	Single	75	22.7
	Married	249	75.5
	Others	6	1.8

Education level	Secondary vocational school	3	0.9
	Junior college	25	7.6
	Undergraduate	296	89.7
	Graduate students and above	6	1.8

Experience (years)	1–5	56	17.0
	6–10	38	11.5
	> 10	236	71.5

Work Units	Internal medicine	172	52.1
	Surgery	85	25.8
	Obstetrics and gynecology	35	10.6
	Pediatrics	26	7.9
	Other Departments	12	3.6

### 3.2. Validity Analysis

#### 3.2.1. Content Validity

The content validity of the NTWQ‐C was determined using the assessments of 10 nursing experts. The results demonstrated that the I‐CVI for each item was between 0.90 and 1.00, with an S‐CVI/Ave of 0.985.

#### 3.2.2. Structural Validity

##### 3.2.2.1. EFA

**FIGURE 1:**
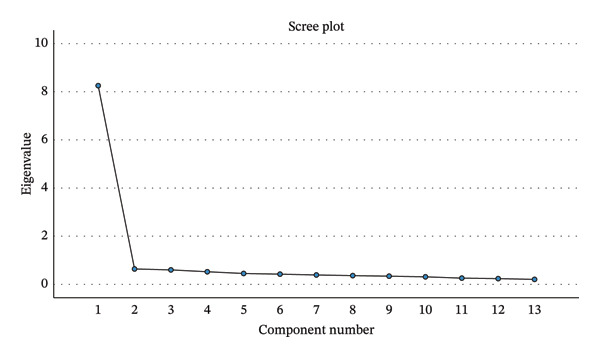
Scree plot of EFA for NTWQ‐C.

The KMO and Bartlett’s test of sphericity showed that the sample data was sufficient for EFA (KMO = 0.960, *χ* ^2^ = 1202.469, df = 78, *p* < 0.001). Principal component analysis and varimax rotation revealed a single factor with eigenvalues ≥ 1.0, which explains 63.481% of the variance. The scree plot supported the single‐factor pattern (Figure 1). All items had factor loadings > 0.40 and were therefore retained.

##### 3.2.2.2. CFA

**FIGURE 2:**
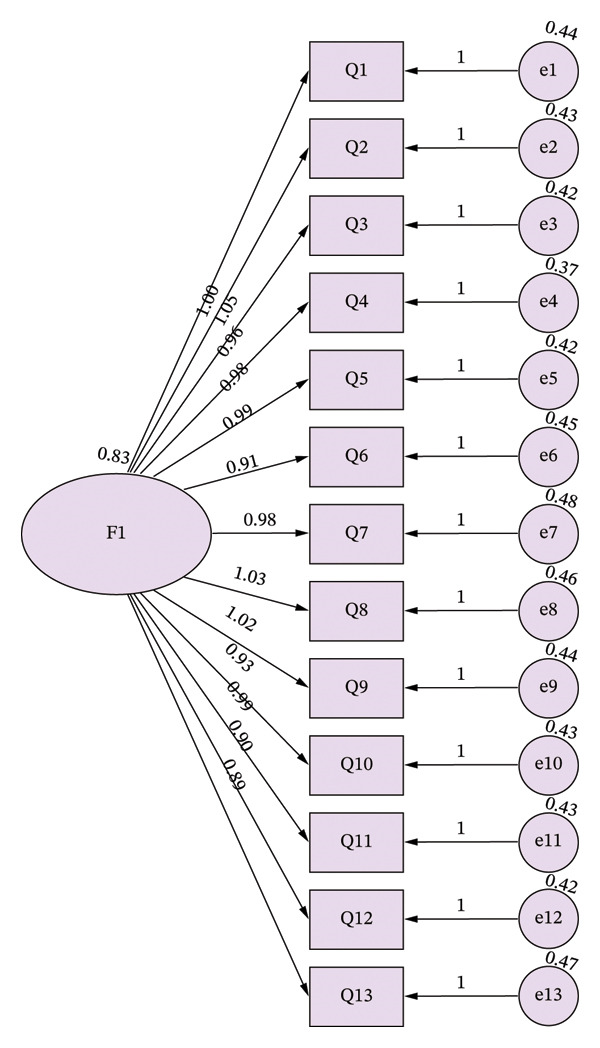
Confirmatory factor analysis for NTWQ‐C.

CFA assessed model fit using *χ* ^2^/df, RMSEA, GFI, TLI, CFI, and IFI. Results indicate good model fit (Table 2). These findings supported the structural validity of the single‐factor, 13‐item model. S1 shows the standardized factor loadings for each item, ranging from 0.765 to 0.826. Figure 2 shows the questionnaire items and the standardized factor loadings.

**TABLE 2:** CFA model fit indices.

Fit index	*χ* ^2^/df	RMSEA	GFI	TLI	CFI	IFI
Reference value	< 3.000	< 0.08	> 0.900	> 0.900	> 0.900	> 0.900
Measured value	1.226	0.034	0.942	0.991	0.993	0.993

All standardized factor loadings exceeded 0.70 (range 0.765–0.826). The AVE was 0.641 and the CR was 0.959, both exceeding the recommended thresholds of 0.50 and 0.70, respectively, indicating satisfactory convergent validity of the NTWQ‐C.

#### 3.2.3. Reliability

##### 3.2.3.1. Internal Consistency

Reliability analysis revealed that the NTWQ‐C has excellent internal consistency, with Cronbach’s alpha of 0.956. To investigate excessive items, we determined the impact of eliminating each item on the total Cronbach’s alpha. The Cronbach’s α‐if‐item‐deleted did not exceed 0.953 for any item, indicating no redundancy; all items were therefore retained. The final version of the Nurses Time‐Wasters Questionnaire is shown in S2.

## 4. Discussion

This study conducted a systematic psychometric evaluation of the NTWQ‐C. Both EFA and CFA supported the original questionnaire’s single‐factor structure, explaining 63.481% of the variance. The model fit indices were favorable (e.g., *χ* ^2^/df = 1.226 and RMSEA = 0.034), meeting psychometric standards [30]. Meanwhile, the questionnaire demonstrated excellent internal consistency reliability (Cronbach’s *α* = 0.956). These findings confirm that the Chinese‐language questionnaire is a structurally sound, accurate, and effective tool for assessing time wasters among Chinese nurses.

This localization strictly follows the cross‐cultural adaptation process recommended by Beaton et al. [26], aiming to ensure the semantic, idiomatic, empirical, and conceptual equivalence of the scale in the context of Chinese culture. We have localized the wording of certain items for better adaptation. For instance, we simplified “mobile phones and social media” in Item 2 to “mobile phones.” This is because, within the Chinese context, mobile phones serve as the primary device encompassing all social applications. This modification preserves the core concept of the original item—using phones and social media for personal matters during working hours—while aligning more closely with the language habits and practical realities of Chinese nurses. All these adjustments underwent expert committee review and pre‐experimental validation, fundamentally ensuring the scale’s accuracy and validity when measuring Chinese populations.

A noteworthy psychometric consideration of this study is that reliability assessment was limited to internal consistency, as measured by Cronbach’s alpha. The obtained coefficient (*α* = 0.956) demonstrates that the items of the NTWQ‐C cohesively measure a single underlying construct at a given point in time. However, internal consistency alone does not capture all dimensions of reliability. Specifically, temporal stability was not evaluated in the present study. Temporal stability refers to the degree to which the instrument yields consistent results when administered to the same respondents on separate occasions [38]. Test‐retest reliability is a critical psychometric property, particularly for an instrument intended to identify time‐wasting factors in clinical practice. Many of the factors assessed by the NTWQ‐C (e.g., workflow inefficiencies, system response times, and individual procrastination tendencies) are expected to remain relatively stable over short intervals in the absence of targeted intervention. Demonstrating temporal stability is therefore essential before the questionnaire can be applied in practice. Potential applications include longitudinal monitoring, evaluation of organizational interventions, and pre–post designs to assess the effectiveness of time‐management training programs. Without test–retest data, it is difficult to determine whether observed changes in scores reflect genuine shifts in time‐wasting behaviors or simply measurement error. Future psychometric work on the NTWQ‐C should prioritize a test–retest study. Ideally, the questionnaire should be administered to the same sample of nurses at a 2‐ to 4‐week interval [39, 40]. Temporal stability can be quantified using the intraclass correlation coefficient (ICC), with values ≥ 0.70 typically considered acceptable [41].

A major advantage of this scale is its systematic assessment perspective. Its 13 items clearly cover time wasters across the individual (e.g., procrastination in decision‐making), interpersonal (e.g., communication with family members), and systemic (e.g., slow HIS response) dimensions. Compared to Zyoud’s scale [8], which primarily focuses on individual behaviors, this assessment offers greater comprehensiveness. It suggests that time management challenges among Chinese nurses represent a systemic issue involving the interplay of multiple factors.

The original development study and the present study characterized the items using different metrics—endorsement frequency (item means) in the former and factor loadings in the latter—so the item‐level results are not directly equivalent: a high mean reflects a frequently occurring behavior, whereas a high loading reflects an item that strongly represents the underlying time‐waster construct. Bearing this distinction in mind, personal phone use was salient in both samples, among the most frequently reported time wasters in the original Palestinian sample (mean = 3.6) and one of the most strongly loading indicators in the present Chinese sample (loading = 0.825), suggesting that it is both a common and a prototypical time waster across settings. Communication with patients′ relatives, visitors, and colleagues showed an equally high loading here (0.826) but was only moderately endorsed in the original sample (*M* = 3.4), indicating that interpersonal interruptions are a comparatively more central feature of time waste among Chinese nurses. In China’s high‐workload nursing environment, frequent interruptions may increase nurses′ stress and contribute to occupational burnout [42, 43]. Beyond these individual and interpersonal sources, the questionnaire also captures system‐level wasters—long waits for doctors′ orders, slow HIS response, and duplicate recording across the HIS and paper documentation—which, although not the most strongly loading items, all loaded acceptably on the single factor (≥ 0.76) and thus belong to the same construct. These directly point to technological system inefficiencies. These findings hold significant practical implications for nursing administrators: interventions should extend beyond individual time management training for nurses. Instead, systemic reforms at the organizational level—such as optimizing workflows and upgrading information systems—are essential to fundamentally improve time utilization.

Time wasters among nurses are not merely a matter of individual time‐management ability but are deeply embedded in the work environment and organizational conditions such as workflow design and resource availability. For instance, the item “searching for misplaced supplies or equipment” reflects inadequate logistical support and resource management rather than personal negligence. Consistent with this view, an adverse work environment characterized by repeated interruptions, heavy communication demands, and inefficient work processes has been shown to elevate nurses’ stress, lower their professional quality of life, and consequently undermine their capacity to organize and manage time effectively [44]. This implies that durable gains in time utilization cannot be secured through individual training alone but require optimization of the work environment and its supporting systems. At the same time, nurses’ personal resources continue to play a buffering role: those with higher emotional intelligence and more adaptive coping behaviors are better equipped to manage the stress generated by demanding clinical environments [45]. A multilevel strategy that couples organizational reform, including streamlining workflows and strengthening supply and information systems, with efforts to enhance nurses’ coping capacity and emotional resilience is likely to be the most effective means of reducing time wasters and alleviating their associated burden.

Therefore, identifying and reducing these time wasters holds urgent clinical significance. Research indicates that frequent time interruptions and inefficient workflows significantly diminish nursing efficiency [46, 47] and increase the risk of medication errors [15] and the level of nursing burnout [48]. Applying this scale to accurately identify key sources of time wasters in clinical settings can provide evidence‐based support for developing targeted intervention strategies. This approach enhances work efficiency, ensures patient safety, and promotes nurses’ physical and mental well‐being.

This study introduces the NTWQ‐C developed by Qtait into the field of Chinese nursing research, filling a gap in domestic assessment tools for identifying factors contributing to nurses’ time wasters. Featuring a simple structure and clear items, the questionnaire facilitates implementation in clinical and management settings. It aids in identifying time‐wasting factors that impact nursing efficiency and provides a basis for developing targeted time management interventions.

### 4.1. Limitations

However, this study still has certain limitations. Although the sample originates from multiple departments, it primarily consists of general wards in internal medicine and surgery. Future research should further expand the sample scope and representativeness, particularly verifying its applicability in high‐pressure clinical settings such as emergency departments and ICUs. Second, several psychometric properties were not examined. Because the NTWQ‐C is a unidimensional instrument, conventional discriminant validity was not applicable. This type of validity typically relies on comparisons among multiple latent factors (e.g., the Fornell–Larcker criterion [32] or the heterotrait–monotrait ratio [49]). In future studies, discriminant validity could be assessed by administering the NTWQ‐C alongside theoretically unrelated measures. Known‐group validity was likewise not formally tested, as no a priori groups with well‐established differences in time‐wasting factors were available; subsequent work could examine whether the instrument distinguishes groups hypothesized to differ, such as nurses in high‐acuity versus general‐ward settings. Then the scale has not yet been validated for criterion validity due to the lack of a gold standard for assessing nurses’ time‐wasting factors. Furthermore, this study employed a cross‐sectional design and did not evaluate the scale’s test–retest reliability. Future research should increase sample diversity, incorporate longitudinal designs to validate predictive validity, and explore the scale’s application in intervention studies.

## 5. Conclusion

The NTWQ‐C comprises one dimension and 13 items, demonstrating strong psychometric properties. It serves as a reliable measurement tool for assessing time‐wasting factors among Chinese nurses. Additionally, managers can utilize this instrument for time planning. It also helps identify nurses’ time‐wasting behaviors and evaluate the effectiveness of related intervention measures.

## Author Contributions

R.W. performed the statistical analysis and wrote the paper; R.W., K.F., and Y.H. contributed to the data collection; J.Z. and C.N. made substantial contributions to the study conception and design; K.F. and C.N. collaborated on the study and revised the manuscript.

## Funding

There is no funding for this study.

## Disclosure

All authors read and approved the final manuscript.

## Ethics Statement

This study was approved by the Ethics Committee of First Affiliated Hospital of China Medical University (NO. 2025–595) and adhered to the ethical principles of the Declaration of Helsinki.

## Consent

The authors have nothing to report.

## Conflicts of Interest

The authors declare no conflicts of interest.

## Supporting Information

Additional supporting information can be found online in the Supporting Information section.

## Supporting information


**Supporting Information S1** Original item, translated item, modified Chinese version and rationale for modification.

**Supporting Information S2** Standardized factor loadings of the NTWQ‐C.

**Supporting Information S3** The final version of the NTWQ‐C.

## Data Availability

The data that support the findings of this study are available from the corresponding author upon reasonable request.
