# Supplementary material for: Chinese Adaptation of the Nurses Time‐Wasters Questionnaire: Reliability and Validity Testing
Source: J Nurs Manag. 2026 Aug 17;2026:2162912. doi: 10.1155/jonm/2162912 (PMC13478914; doi:10.1155/jonm/2162912)
Supplement: Supplementary file 1 [file JONM-2026-2162912-s001.docx]

S3. The final version of the NTWQ-C

| 序号(NO.) | 条目  (Items) | 从不(Never) | 很少(Rarely) | 有时(Sometimes) | 经常(Often) | 总是(Always) |
| --- | --- | --- | --- | --- | --- | --- |
| 1 | 由于护士站和病室之间的距离问题，频繁地往返，我认为可能会浪费时间。 |  |  |  |  |  |
| 2 | 在工作时间内使用手机处理个人事务。 |  |  |  |  |  |
| 3 | 等待医生下达医嘱时间较长。 |  |  |  |  |  |
| 4 | 与患者的家属、探视者或同事闲聊。 |  |  |  |  |  |
| 5 | 寻找未归位的用品或设备。 |  |  |  |  |  |
| 6 | 因能力不足导致护理任务不能按时完成。 |  |  |  |  |  |
| 7 | 拖延完成那些我不喜欢但又必须完成的任务。 |  |  |  |  |  |
| 8 | 制定决策时拖延。 |  |  |  |  |  |
| 9 | 低估任务所需时间。 |  |  |  |  |  |
| 10 | 与其他部门协调患者需求、护理及随访工作时不顺畅。 |  |  |  |  |  |
| 11 | 手动填写护理文书。 |  |  |  |  |  |
| 12 | 医院信息系统（HIS）反应迟钝。 |  |  |  |  |  |
| 13 | 一项护理任务需要同时在电子健康信息系统和护理文书上记录。 |  |  |  |  |  |
