# Supplementary material for: Chinese Adaptation of the Nurses Time‐Wasters Questionnaire: Reliability and Validity Testing
Source: J Nurs Manag. 2026 Aug 17;2026:2162912. doi: 10.1155/jonm/2162912 (PMC13478914; doi:10.1155/jonm/2162912)
Supplement: Supplementary file 2 [file JONM-2026-2162912-s002.docx]

S1. Original item, translated item, modified Chinese version and rationale for modification.

|  | Original item | Translated item | Modified Chinese version | Rationale for modification |
| --- | --- | --- | --- | --- |
| 1 | Moving around between patient’s rooms, nursing station and rest room may delay the required tasks | 护士在休息室、护士站与病房的移动可能会耽误需要做的工作。 | 由于护士站和病室之间的距离问题，频繁地往返，我认为可能会浪费时间。 | During the forward and back-translation process, we discovered that the entry was open to interpretation. After the research team discussed the issue and sought clarification from the original author, we expanded the content of the entry. “Moving around” refers not only to distance but also to the frequency of movement. |
| 2 | Using phones and social media for personal matters during working hours. | 在工作时间内使用手机和社交软件处理个人事务。 | 在工作时间内使用手机处理个人事务。 | In China, the mobile phone included social media apps that nurses use on a daily basis, so it was determined that these apps were redundant and were deleted. |
| 3 | Waiting physicians to receive medication orders, post-round information, discharge orders and other necessary information. | 等待接收医生发出的用药指令、查房后病人的信息、出院医嘱和其他必要信息。 | 等待医生下达医嘱时间较长。 | In China’s healthcare system, medication orders, post-round information, discharge orders, and other necessary instructions are all included in the medical orders issued by doctors. Following discussions with experts and confirmation from the original author, the sentence was revised to read: “Long waiting times for doctors’ orders.” |
| 5 | Searching for supplies or equipment to be used and then bring them back. | 寻找要使用的用品或设备，并将其带回。 | 寻找未归位的用品或设备。 | Following expert discussion and in light of clinical nursing practice, all supplies and equipment—whether currently in use or not—must be returned to their designated locations. Therefore, the statement has been revised to read: “Searching for misplaced supplies or equipment.” |
| 6 | Non competence and delay in getting nursing task done on time | 没有能力不能按时完成护理任务。 | 因能力不足导致护理任务不能按时完成。 | The original translation did not conform to standard Chinese usage, so it has been revised. |
| 10 | Coordination among health professional for patients need, care and follow-up | 为满足患者需求、护理及随访而与其他卫生专业人员进行的协调工作。 | 与其他部门协调患者需求、护理及随访工作时不顺畅。 | The original translation did not conform to standard Chinese usage, and experts unanimously agreed that it was ambiguous when read. After consulting the author regarding their understanding of the entry, the entry was revised. |
| 11 | Manual charting and documentation | 手动绘制图表和书写护理文书。 | 手动填写护理文书。 | In the practice of clinical nursing in China, charts are also classified as nursing documentation. Experts believe that the charts listed here duplicate the nursing documentation, so they have been removed. |
| 12 | Frequent disorder of Hospital information system (HIS) | 医院信息系统（HIS）经常失灵。 | 医院信息系统（HIS）反应迟钝。 | Experts unanimously agree that the statement “Frequent disorder of Hospital information system” does not reflect the actual clinical situation in China; “slow response” is more consistent with the reality in Chinese hospitals. Therefore, the statement has been revised to “Slow response from the Hospital Information System (HIS).” |
| 13 | Utilizing of use both system (manual and health information system | 同时使用两种系统（护理文书书写和电子健康信息系统） | 一项护理任务需要同时在电子健康信息系统和护理文书上记录。 | Experts determined that the original translation did not conform to standard Chinese usage. After consulting the original author regarding their interpretation of the entry, it was revised to read: “A single nursing task requires simultaneous recording in both the health information system and nursing documentation.” |
