# Supplementary material for: Chinese Adaptation of the Nurses Time‐Wasters Questionnaire: Reliability and Validity Testing
Source: J Nurs Manag. 2026 Aug 17;2026:2162912. doi: 10.1155/jonm/2162912 (PMC13478914; doi:10.1155/jonm/2162912)
Supplement: Supplementary file 3 [file JONM-2026-2162912-s003.docx]

S2. Standardized factor loadings of the NTWQ-C

| Items | Factor loading |
| --- | --- |
| The distance between the nursing station and patient rooms makes frequent trips inefficient. | 0.807 |
| Using phones for personal matters during working hours. | 0.825 |
| Long waiting times for doctors’ orders. | 0.802 |
| Communication with patient relatives, visitors, and colleagues. | 0.826 |
| Searching for misplaced supplies or equipment. | 0.812 |
| Failure to complete nursing tasks on time due to insufficient competence. | 0.777 |
| Procrastination on tasks that I don’t like but it must be done. | 0.789 |
| Procrastination in decision-making | 0.809 |
| Underestimating time for tasks. | 0.814 |
| Coordination of patient needs, care, and follow-up with other departments is not smooth. | 0.790 |
| Manually filling out nursing documentation. | 0.808 |
| Slow response from the Hospital Information System (HIS). | 0.785 |
| A single nursing task requires simultaneous recording in both the health information system and nursing documentation. | 0.765 |
